# Quantitation of pesticides in bee bread collected from honey bee colonies in an agricultural environment in Switzerland

**DOI:** 10.1007/s11356-023-26268-y

**Published:** 2023-03-14

**Authors:** Emmanuel Schaad, Marion Fracheboud, Benoît Droz, Christina Kast

**Affiliations:** Swiss Bee Research Centre, Agroscope, Schwarzenburgstrasse 161, 3003 Bern, Switzerland

**Keywords:** *Apis mellifera*, Bee bread, Pollen, Residues, Pesticides, QuEChERS, UHPLC-MS/MS

## Abstract

Pesticide contamination of bee products is a widespread phenomenon. Due to its composition, bee bread is affected by both lipophilic and hydrophilic substances. As proof of concept of a monitoring campaign and to better understand the extent of contamination, we developed an analytical method based on a modified QuEChERS extraction, with subsequent separation by liquid chromatography and detection by mass spectrometry. This allowed for the quantitation of 51 agricultural- or beekeeping-associated pesticides in bee bread. The workflow was applied to 60 samples taken biweekly throughout spring to autumn 2022 from five colonies at a Swiss apiary in an agricultural area. In total, 30 pesticides were identified (> LOD), among which 26 pesticides were quantitated. The total number of pesticides detected per colony ranged from 11 to 19. The most prevalent substances (> LOQ) were two neonicotinoid insecticides, acetamiprid and thiacloprid (max. 16 μg/kg and 37 μg/kg, respectively); seven fungicides, azoxystrobin (max. 72 μg/kg), boscalid (max. 50 μg/kg), cyprodinil (max. 1965 μg/kg), difenoconazole (max. 73 μg/kg), mandipropamid (max. 33 μg/kg), pyraclostrobin (max. 8 μg/kg) and trifloxystrobin (max. 38 μg/kg); and two herbicides, prosulfocarb (max. 38 μg/kg) and terbuthylazine (max. 26 μg/kg). The study revealed strong variability in pesticide occurrence and concentrations among colonies sampled at the same site and date. The applied biweekly sampling of bee bread from March to August was shown to be reliable in capturing peak contaminations and revealing the onset of certain pesticides in bee bread. The study provides an adequate practical approach for pesticide monitoring campaigns.

## Introduction

Agriculture and floral ecosystems depend highly on honey bees (*Apis mellifera*) as pollinators. Honey bees commonly forage within a distance of 2 km, depending on the availability of food sources, and rarely fly more than 6 km (Visscher & Seeley, 1982). While foraging, honey bees are exposed to a plethora of environmental contaminants, including pesticides from agricultural activities (Murcia-Morales et al., 2022). When bees collect nectar, pollen, or water, they may also bring contaminants into their hives (Bogdanov, 2005). In this context, bee products collected by honey bees can serve for biomonitoring the foraging area with respect to environmental contaminants (van der Steen 2016).

Many studies have reported pesticide contamination of bee products (Beyer et al., 2018; Bokšová et al., 2021; Daniele et al., 2018; Orantes-Bermejo et al., 2015). Pesticides are frequently found in pollen (e.g., Friedle et al., 2021), beeswax (e.g., Marti et al., 2022; Wilmart et al., 2021), or honey (e.g., Souza Tette et al., 2016). Within the various hive compartments, lipophilic pesticides with a high logarithmic octanol-water partition coefficient tend to accumulate in beeswax. Over the past 30 years, Swiss beeswax has been tested for beekeeping-associated, lipophilic pesticides as part of the efforts to maintain good wax quality and, consequently, minimize the exposure of honey bees to pesticides in beeswax. Such monitoring has proven useful to prevent high levels of lipophilic pesticides in beeswax (Kast et al., 2021). However, a large variety of pesticides — both lipophilic and hydrophilic contaminants with a broad range of logarithmic octanol-water partition coefficients — may be primarily present in fresh or stored pollen (bee bread) (Murcia Morales et al., 2020). Hence, it is advantageous to include bee bread in a long-term monitoring program.

Pollen and bee bread serve as protein sources for larvae and newly emerged honey bees. Lack of pollen or low quality of pollen affects worker longevity (Di Pasquale et al., 2016). Furthermore, the development of the hypopharyngeal glands in young bees required later to produce larval jelly is highly dependent on protein intake (Di Pasquale et al., 2016), and nurse bees consume pollen and bee bread to provide a protein-rich jelly for larvae. Finally, well-fed larvae are a prerequisite for the rearing of healthy, long-lived winter bees, which are crucial for colony survival during winter months (Locke et al., 2014). Honey bees store part of the collected pollen as bee bread in the combs for later consumption. For storage, pollen is biochemically processed by enzymes derived from the saliva and gastric fluid of the honey bees (Giroud et al., 2013). Depending on brood presence and pollen availability in the environment, the amount of stored pollen may differ (Roessink & van der Steen, 2021). Field studies have shown that honey bees consume stored pollen (bee bread) preferably within a few days after collection (Carroll et al., 2017). Roessink and van der Steen (2021) demonstrated that 70% of bee bread is consumed within the first 5 days, whereas the remainder is consumed over 2 to 3 weeks. Therefore, little pollen older than 2 weeks is usually expected in bee bread at any given time during spring and summer. Hence, the analysis of bee bread should cover a collection period of up to two weeks. Sampling every second week most likely ensures a sufficient turnover of bee bread and so the bee bread predominantly includes pollen collected during the 2 weeks preceding the sampling. Based on this, bee bread was selected in this study as the matrix in order to cover a wide range of pesticides.

In an agricultural environment, honey bees may collect pollen and nectar from mass-flowering crops, like oilseed rape (OSR) and sunflowers, as well as pollen from maize (Requier et al., 2015). As OSR and sunflower are entomophilous taxa, they are dependent on pollination by honey bees. While these crops, along with maize, are attractive to honey bees, they are also often treated with pesticides, leading to the exposure of foraging bees to these substances. Consequently, these three taxa are the most researched plants in connection to neonicotinoid residues from agricultural practices impacting bees (Lundin et al., 2015).

Recently, efforts have been made to reduce the risk associated with the use of pesticides. In 2017, the Swiss government adopted an action plan with the aim to reduce the risk by 50% by the year 2027, using the years 2012–2015 as a reference period. Simultaneously, alternatives to chemical crop protection are being promoted and supported (Swiss Government, 2017, 2021). Hence, monitoring pesticides in bee bread might be useful to study the effectiveness of these political decisions.

The legislation in place regarding the allowance and use of pesticides in agricultural practices differs from one country to the other. Therefore, it is important to study contamination levels in each individual region of interest. Thus, our aim was to establish a method to monitor a variety of pesticides by analysing bee bread collected at an apiary in Switzerland. For this purpose, appropriate analytical methods were developed for the quantitation of a range of lipophilic as well as hydrophilic pesticides in bee bread. Firstly, sensitive, analytical methods for 51 pesticides were validated. Secondly, an apiary in an agricultural environment was chosen to evaluate ideal sampling time points, sampling frequency, and the minimal number of colonies needed to produce a robust temporal record of pesticide contamination in bee bread.

## Material and methods

### Chemicals and utilities

The following reference standards were obtained from LGC Standards GmbH (Wesel, Germany): acetamiprid (C10013000), aclonifen (C10042000), azoxystrobin (C10413000), bendiocarb (C10460000), boscalid (C10663000), bromopropylate (C10762000), chlorfenvinphos (C11290000), chlorpyrifos (C11600000), clothianidin (C11691700), clothianidin-D3 (C11691710), lambda-cyhalothrin (C11860000), zeta-cypermethrin (C11890500), cyproconazole (C11908000), cyprodinil (C11909000), deltamethrin (C12120000), dimethoate (C12700000), N-(2,4-dimethylphenyl)formamide (DMF) (C12737000), difenoconazole (C12609000), dimoxystrobin (C12775000), fenhexamid (C13476000), fenitrothion (C13480000), (E)-fenpyroximate (C13545000), fludioxonil (C13705000), flufenacet (C13711000), tau-fluvalinate (C13870000), flumethrin (C13719000), fluopyram (C13743000), hexythiazox (C14210000), imidacloprid (C14283700), indoxacarb (C14325500), iprovalicarb (C14371000), mandipropamid (C14745000), mepanipyrim (C14867000), metconazole (C14955000), methoxyfenozide (C15080500), permethrin (C15990000), piperonyl butoxide (C16240000), propoxur (C16500000), prosulfocarb (C16545000), desthio-prothioconazole (C16555500), pyraclostrobin (C16595000), spirodiclofen (C16972950), tebuconazole (C17178700), terbuthylazine (C17300000), thiamethoxam (C17453000), and trifloxystrobin (C17842000). Additionally, acrinathrin (46415), coumaphos (45403), flupyradifurone (37050), and thiacloprid (37905) were purchased from Sigma-Aldrich (Buchs, Switzerland), while fipronil (15900-2365-10AN10) was obtained from NEOCHEMA (Bodenheim, Germany).

Acetonitrile SupraSolv (1.00017) and 2-propanol (1.01040), LiChroSolv quality, were purchased from Merck (Darmstadt, Germany). Formic acid solution 50% for HPLC (09676) was acquired from Honeywell Fluka (Buchs, Switzerland) and ammonium formate (70221) from Supelco (Darmstadt, Germany). The calibrant mix G1969-85000 was obtained as tune solution from Agilent (Santa Clara, California, USA).

For the extraction process of pesticides in bee bread, the following substances were used: magnesium-sulfate (63136) and sodium hydrogencitrate sesquihydrate (359084) from Sigma-Aldrich (Buchs, Switzerland), sodium chloride (1.06404) and tri-sodium citrate dehydrate (1.06448.0500) from Merck (Darmstadt, Germany), and Bondesil PSA 40 μm (12213024), and Bondesil C18 40 μm (12213012) from Agilent Technologies (Santa Clara, California, USA).

A total of 1.5 mL Eppendorf Safe-Lock tubes (11.3119.06), 1.5 mL auto-sampler vials (190804.00), 2 mL single-use syringes (3.7410.02), and single-use polyamide filter (12.3663.01) were obtained from Huberlab (Aesch, Switzerland). Polystyrene Petri dishes (633180) and 50 mL polypropylene (PP) centrifugation tubes (227270) were purchased from Greiner Bio (St. Gallen, Switzerland).

### Bee bread serving as a blank

The bee bread used as a blank extract or for spiking the pesticides to obtain recovery values was collected in 2015 and 2017 from several honey bee colonies owned by Agroscope, located in Liebefeld, Switzerland (46°55′46.9″N 7°25′26.8″E). In the urban environment, the overall contamination level of pesticides was low. Nevertheless, the bee bread contained azoxystrobin, trifloxystrobin, and difenoconazole at a concentration of approximately 3, 2, and 10 μg/kg, respectively.

### Study site and honey bee colonies

The honey bee (*Apis mellifera*) colonies used in this study were located in an agricultural region in Northwestern Switzerland (46°58′57.6″N, 7°08′40.2″E). The site was chosen because it is surrounded by agricultural crops with cultivations of OSR, maize, sunflowers, and various vegetables where pesticide application is strongly expected. The occurrence and flowering periods of OSR, maize, and sunflower were noted during the sampling period, since they are attractive crops for honey bees. The colonies were treated against *Varroa destructor* infestation using organic acids the year before in August and December 2021. All colonies were overwintered in 12-frame Dadant-Blatt hives (the number of frames was reduced to 7 to 8 during winter), with combs up to about 3 years old. In 2022, treatment against *V. destructor* infestation was performed in four colonies, from 19th to 31st August, using a Nassenheider Pro (290 mL formic acid 60%, wick 2), while treatment was not necessary for one of the colonies. The colonies were fed with 5–7 L of syrup (60 % sugar) from 22nd July to 6th September 2022.

### Bee bread sampling

Five colonies were selected based on a first inspection regarding the availability and quantity of stored bee bread in early spring. The first sampling served as a control, since it took place at the end of winter on 29th March 2022. Subsequently, samples were taken every second week from 15th April until 18th August 2022 (11 sampling dates) to cover a whole crop season. After formic acid treatment, a final sampling took place on 4th October 2022.

Whenever possible, two suitable combs containing fresh bee bread were chosen from each colony. From each comb, one rectangle (approximately 30 cm^2^) was cut out using a clean knife. The samples were stored at −20 °C until further use. To separate the bee bread from the comb, a tool designed by Gürle Aricilik (Nilüfer Bursa, Turkey) was used (Ürünler | gurlearicilik). It consists of a metal plunger with a spring that extracts the content of a cell. The bee bread from the two comb pieces of the same colony collected at each sampling date was combined and subsequently homogenized in a petri dish using a custom 3D-printed pestle (produced at Agroscope), resulting in sample weights of 5 to 15 g of bee bread. This procedure resulted in a total of 60 samples.

### Extraction of pesticides

The extraction of pesticides followed a modified QuEChERS (quick, easy, cheap, efficient, rugged, safe) method, which was based on the procedure described by Niell et al. (2014). PSA and C18 sorbents were used for clean-up. Accordingly, 1 g of bee bread was weighed into a 50-mL PP centrifugation tube, 1 mL of MilliQ water was added, and then, the bee bread was suspended using a Vortex-Genie 2 mixer from Scientific Industries (New York, USA). After 15 min of resting, 4 mL of acetonitrile containing 50 μg/L clothianidin-D3 was added. The tubes were then vigorously shaken by hand three times for 30 s, with 15 min of rest in-between. Subsequently, a salt kit containing 0.6 magnesium sulphate, 0.2 g sodium chloride, 0.25 tri-sodium citrate dehydrate, and 0.12 g sodium hydrogencitrate sesquihydrate was added to salt out the aqueous phase. Next, the tubes were shaken for 10 min, using a UNIMAX 2010 sample shaker by Heidolph (Schwabach, Germany), to homogenize the samples. The tubes were then placed in the freezer at −20 °C for 1 h to freeze out the lipid and wax components. Subsequently, the samples were centrifuged at 4 °C and 10,000 g using a Sigma 4-16KS (Osterode am Harz, Germany). Afterwards, three 1 mL aliquots were pipetted into three separate 1.5 mL Safe-Lock tubes. One tube contained 50 mg Bondesil PSA, another contained 50 mg Bondesil C18, and the third one contained both 50 mg Bondesil PSA and 50 mg Bondesil C18. This separation was done since recoveries for cyprodinil and spinosad were found to be higher using only PSA, while using only C18 resulted in enhanced recoveries for fenhexamid and spirodiclofen. All other pesticides were extracted using both PSA and C18. The samples were then vortexed twice for 30 s. Subsequently, the samples were centrifuged at room temperature for 20 min using a Centrifuge 5804 from Eppendorf (Hamburg, Germany). The resulting supernatant was directly filtered into a 1.5-mL auto-sampler vial using a 2-mL single-use syringe coupled with a single-use polyamide filter of pore size 0.45 μm, before analysis with ultra-high-performance liquid chromatography (UHPLC) coupled to mass spectrometry (MS/MS).

### UHPLC-MS/MS analysis

For the analysis of 51 pesticides, three methods (M1, M2, M3) with variable eluent gradients and ion source conditions were established. Liquid chromatography (LC) was performed using an Agilent 1290 Infinity II equipped with an autosampler and coupled with an Agilent 6495C tandem quadrupole mass spectrometer (MS). Chromatographic separation was performed at 40 °C on a C18 reverse phase column (Acquity UPLC HSS T3 Column, 100 Å, 1.8 μm, 2.1 mm × 100 mm) from Waters (Milford, Massachusetts, USA). The temperature of the autosampler was 10 °C, and the injection volume was 1 μL. The mobile phase A was 95% water + 5% acetonitrile + 0.01% formic acid + 5 mM ammonium formate, and the mobile phase B was 5% water + 95% acetonitrile + 0.01% formic acid + 5 mM ammonium formate. The LC gradient conditions for the three methods are shown in Table 1. The column flow was set to 0.5 mL/min. The solvents 2-propanol, acetonitrile, and 0.01 % formic acid in water were used as needle wash.

**Table 1:** Liquid chromatography gradients of methods M1, M2, and M3 used for the quantification of pesticide in bee bread

Step	M1		M2		M3	
	Time (min)	*B*^1^ (v%)	Time (min)	*B*^1^ (v%)	Time (min)	*B*^1^ (v%)
1	0.25	0	0.25	0	0.25	0
2	6.60	80	6.60	100	4.00	40
3	13.00	100	12.00	100	6.60	100
4	13.01	0	12.01	0	9.01	0

^1^The amount of mobile phase A results by subtracting the amount of B from 100%

The detection and quantification of the pesticides were performed on an Agilent 6495C series tandem quadrupole MS system operating in electrospray ionization MS. The ion source conditions of the three methods are listed in Table 2. Methods 1 and 3 operated only in positive ionization mode, while method 2 included both positive and negative ionization modes (negative mode for fibronil). Quantitation and identification were based on selected ion transitions, thereby using one transition for quantitation and two additional transitions for identification. The ion transitions are listed in Table 3.

**Table 2:** Ion source conditions for the individual methods M1, M2, and M3

Condition	M1	M2	M3
Gas temperature (°C)	130	180	250
Gas flow (L/min)	20	20	20
Sheath gas temp (°C)	120	150	320
Sheath gas flow (L/min)	10	6	10
Nebulizer (psi)	30	30	60
Capillary positive (V)	6000	6000	6000
Capillary negative (V)	-	3500	-
Nozzle voltage positive (V)	2000	2000	2000
Nozzle voltage negative (V)	-	0	-

**Table 3:** Selected ion transitions used for qualification and quantification in each method

Analyte	Method		Quantifier		Qualifier 1		Qualifier 2	
		Precursor ion (m/z)	Product ion (m/z)	CE^a^ (V)	Product ion (m/z)	CE^a^ (V)	Product ion (mz)	CE^a^ (V)
Acetamiprid	M3	223.1	56.1	14	99	46	90	42
Aclonifen	M1	265	80	38	194	22	51.1	80
Acrinathrin	M1	559.2	208.2	14	181.2	38	83.2	18
Azoxystrobin	M3	404.1	372.3	14	344.3	26	329.3	34
Bendiocarb	M3	224.1	167.2	6	109.1	18	81.2	42
Boscalid	M3	343	271.3	38	272.3	34	140.1	18
Bromopropylate	M2	444	208.9	42	408.7	6	152.9	66
Chlorfenvinphos	M3	359	155.1	10	205.1	22	170.1	50
Chlorpyrifos	M1	349.9	198	18	125.1	18	97.1	34
Clothianidin	M3	250	113	30	110	30	71	42
Clothianidin-D3	M1-M3	253	131.9	18	-	-	-	-
Coumaphos	M2	363	226.8	30	306.7	18	210.8	34
lambda-Cyhalothrin	M1	467.1	225	14	450.1	6	141	58
zeta-Cypermethrin	M1	433.1	191.1	14	416.3	6	127.1	34
Cyproconazole	M3	292.1	70	16	125	32	89	60
Cyprodinil	M3	226.1	77	66	108	30	39.1	80
Deltamethrin	M1	521	279.1	14	504.1	6	172.1	34
Difenoconazole	M3	406.1	251	30	337	18	188	54
Dimethoate	M3	230	125	16	198.8	0	79	32
Dimoxystrobin	M3	327.2	205	10	238.1	10	89	70
DMF	M3	150.1	106	38	106.9	22	77	50
Fenhexamid	M3	302.1	97.1	26	55.1	46	29.2	80
Fenitrothion	M1	278	246.2	18	109.1	18	125.1	22
(E)-Fenpyroximate	M3	422.2	366.4	18	138.1	34	135.1	34
Fipronil	M2	435	330	12	250	28	183	40
Fludioxonil	M1	266.1	229	6	185	26	158	38
Flufenacet	M1	364.1	152	22	194.1	10	77	80
Flumethrin	M1	527.1	266.8	14	509.9	6	238.8	22
Fluopyram	M3	397.1	173	34	145	70	95	80
Flupyradifurone	M3	289.1	126.1	22	90.1	50	73.1	80
tau-Fluvalinate	M2	503.1	180.9	38	207.9	10	151.9	80
Hexythiazox	M3	353.1	168.1	24	227.9	8	151	32
Imidacloprid	M3	256.1	175.1	14	209	18	84	22
Indoxacarb	M3	528.1	218	22	161.9	62	56.1	42
Iprovalicarb	M3	321.2	119	16	91.1	56	202.9	0
Mandipropamid	M3	412.1	328.1	8	356.1	4	125	40
Mepanipirym	M3	224.1	106.1	24	77	40	209	36
Metconazole	M3	320.1	70.1	24	125	48	89	60
Methoxyfenozide	M1	369.2	149	10	313.1	0	133	24
Permethrin	M1	408.1	183.2	6	355.3	6	165.1	54
Piperonyl butoxide	M3	356.2	177.2	14	119.2	42	91.2	62
Propoxur	M3	210.1	111.1	14	168.2	2	93.1	26
Prosulfocarb	M3	252.1	91.2	20	65.1	60	43.1	16
Desthio-Prothioconazole	M3	312.1	70	20	125	40	89.1	80
Pyraclostrobin	M3	388.1	193.8	8	164.1	12	163.1	20
Spinosad (Spinosyn A)	M3	732.1	142.1	30	98.1	80	97	74
Spinosad (Spinosyn D)	M3	746.5	142	30	98.1	80	97.3	78
Spirodiclofen	M3	411	71	13	295	35	313.1	15
Tebuconazole	M1	308.1	70	40	124.9	47	59	36
Terbuthylazine	M3	230.1	174.1	15	132	25	104	35
Thiacloprid	M3	253	126.1	22	90.2	46	73.1	78
Thiamethoxam	M3	292	211.1	8	181.1	20	132	24
Trifloxystrobin	M3	409.1	186	12	206.1	8	145	52

^a^Collision energy

External matrix-matched calibration with nine concentration levels, ranging from 0.05 μg/L to 1000 μg/L, was used for quantitation. The sample concentrations were calculated based on the linear regression (1/*x*) of the calibration samples. All calculations were performed in Agilent MassHunter quantitative software Version B.08.00 (Basel, Switzerland).

The deuterated substance clothianidin-D3 served as an internal standard to monitor the extraction performance. Furthermore, it served as a visual injection control for all pesticides, but no correctional factor was applied. The limit of detection (LOD) levels were experimentally determined for each pesticide by diluting spiked blank extracts (signal to noise ratio (s/n) at least 3:1). Recoveries were assessed for each pesticide at four to eight spiking levels, ranging from 0.2 to 10,000 μg/kg, with at least five repetitions per spiking level. Recoveries of the spiking levels were between 75 and 125% (except recovery of 69% for lowest spike level of tebuconazole at 5 μg/kg). The lowest spiking level of an individual pesticide, which showed a recovery of at least 75%, was set as its limit of quantification (LOQ) (except tebuconazole). The resulting LOD and LOQ values, including the validated concentration range, are given in Table 4. To survey the long-term consistency of instrument precision and accuracy, control samples with pesticide concentrations of 20 μg/kg or 1000 μg/were measured with each series.

**Table 4:** Validated range for all pesticides together with sensitivity parameters

Analyte	Class	log K_ow_	LOD (μg/kg)	LOQ (μg/kg)	Validated range (μg/kg)
Acetamiprid	i	0.8^a^	0.4	1	1–10,000
Aclonifen	h	4.4^a^	5	5	5–10,000
Acrinathrin	a, i	6.3^a^	8	10	10–10,000
Azoxystrobin	f	2.5^a^	n.a.	10	10–10,000
Bendiocarb	i	1.7^a^	0.4	1	1–10,000
Boscalid	f	3.0^a^	8	10	10–10,000
Bromopropylate	a	5.4^a^	40	50	50–10,000
Chlorpyrifos	i	4.7^a^	2	10	10–10,000
Chlorfenvinphos	i	3.8^a^	0.4	2	2–10,000
Clothianidin	i	0.9^a^	2	2	2–10,000
Coumaphos	a, i	4.1^a^	2	5	5–10,000
lambda-Cyhalothrin	i	5.5^a^	20	20	20–10,000
zeta-Cypermethrin	i	6.6^a^	20	20	20–10,000
Cyproconazole	f	3.1^a^	0.4	2.5	2.5–1000
Cyprodinil	f	4.0^a^	0.4	2	2–10,000
Deltamethrin	i	4.6^a^	40	100	100–10,000
Difenoconazole	f	4.4^a^	n.a.	10	10–10,000
Dimethoate	a, i	0.8^a^	0.5	1	1–1000
Dimoxystrobin	f	3.6^b^	0.4	1	1–10,000
DMF	TP	1.5^a^	1	2	2–10,000
Fenhexamid	f	3.5^a^	4	5	5–10,000
Fenitrothion	i	3.3^a^	4	5	5–10,000
(E)-Fenpyroxymate	a	5.7^a^	0.8	2	2–10,000
Fipronil	i	4.0^c^	0.2	0.2	0.2–100
Fludioxonil	f	4.1^a^	2	10	10–10,000
Flufenacet	h	3.5^d^	0.4	1	1–10,000
Flumethrin	i	6.2^a^	2	5	5–10,000
Fluopyram	f	3.3^a^	0.4	2	2–10,000
Flupyradifurone	i	1.2^a^	0.4	1	1–10,000
tau-Fluvalinate	i	7.0^a^	2	5	5–10,000
Hexythiazox	a	2.7^a^	0.8	2.5	2.5–1000
Imidacloprid	i	0.6^a^	2	5	5–10,000
Indoxacarb	i	4.7^a^	0.4	5	5–10,000
Iprovalicarb	f	3.2^a^	0.8	1	1–1000
Mandipropamid	f	3.2^a^	2	2	2–10,000
Mepanipyrim	f	3.3^a^	0.8	1	1–1000
Metconazol	f	3.9^a^	2	5	5–10,000
Methoxyfenozide	i	3.7^a^	0.4	0.5	0.5–1000
Permethrin	i	6.1^a^	4	5	5–10,000
Piperonyl butoxide	s	4.8^a^	2	2	2–10,000
Propoxur	a, i	0.1^a^	0.4	1	1–10,000
Prosulfocarb	h	4.5^a^	0.4	2	2–10,000
Desthio-Prothioconazole	TP	3.0^a^	0.4	5	5–1000
Pyraclostrobin	f	4.0^a^	0.4	2	2–10,000
Spinosad^f^	i	4.0^d^, 4.5^e^	2	5	5–10,000.
Spirodiclofen	a	5.8^a^	2	2.5	2.5–1000
Tebuconazole	f	3.7^a^	2	5	5–10,000
Terbuthylazine	h	3.4^a^	0.4	0.5	0.5–10,000
Thiacloprid	i	1.26^a^	2	5	5–10,000
Thiamethoxam	i	−0.1^a^	0.4	2	2–10,000
Trifloxystrobin	f	4.5^a^	n.a.	5	5–10,000

^a^Lewis et al. (2016)

^b^PubChem (2022a)

^c^PubChem (2022b)

^d^Spinosyn A: Environmental Protection Agency (1997)

^e^Spinosyn D: Environmental Protection Agency (1997)

^f^Spinosad is composed of spinosyn A and spinosyn D. Spinosad was present in a 84:16 concentration mixture in the study at hand

Some pesticides were found in the blank bee bread, including azoxystrobin, trifloxystrobin, and difenoconazole at concentrations of approximately 3, 2, and 10 μg/kg, respectively. Therefore, the LOQs for these pesticides were set accordingly to 10, 5, and 10 μg/kg, respectively, while the LODs for these compounds were not determined (Table 4).

## Results

An analytical procedure was validated, allowing for the quantitation of 51 pesticides in bee bread (Table 4). High sensitivity was achieved for 47 of the tested pesticides, with LOQs ranging between 0.2 and 10 μg/kg at recovery rates above 75% at the corresponding LOQ levels (except tebuconazole). The described analytical procedure was less sensitive for four of the tested pesticides with LOQs ranging from 20 to 100 μg/kg (bromopropylate, lambda-cyhalothrin, zeta-cypermethrin, deltamethrin).

**Fig. 1:**
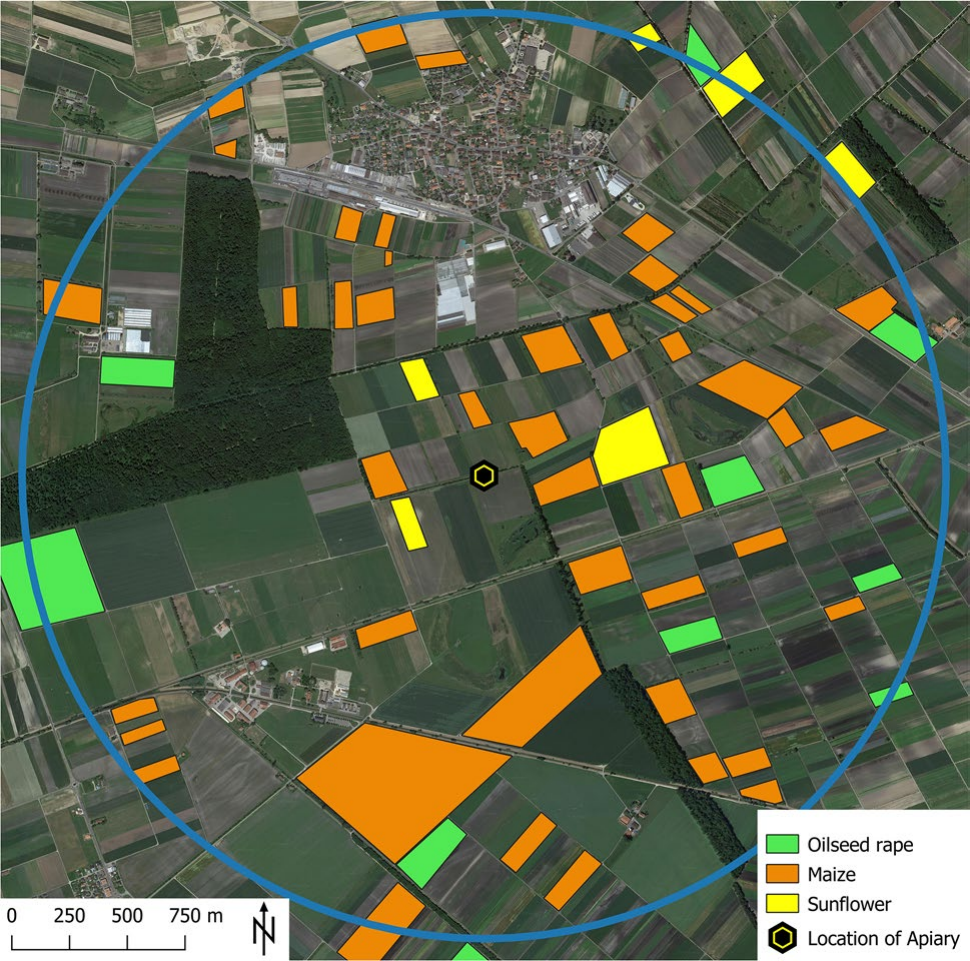
Location of the study apiary with mappings of oilseed rape (OSR), maize, and sunflower cultivations during the year 2022 within a 2-km radius around the study apiary (blue circle)

The cultivations of OSR, maize, and sunflower were mapped within a 2-km radius around the apiary during the crop season 2022 (Fig. 1). Here, maize fields made up the largest fraction among these three crop types, followed by OSR and sunflowers. The flowering periods were observed to be from 5th April to 15th May (OSR), 1st July to 1st September (maize), and 1st July to 25th July (sunflower).

**Fig. 2:**
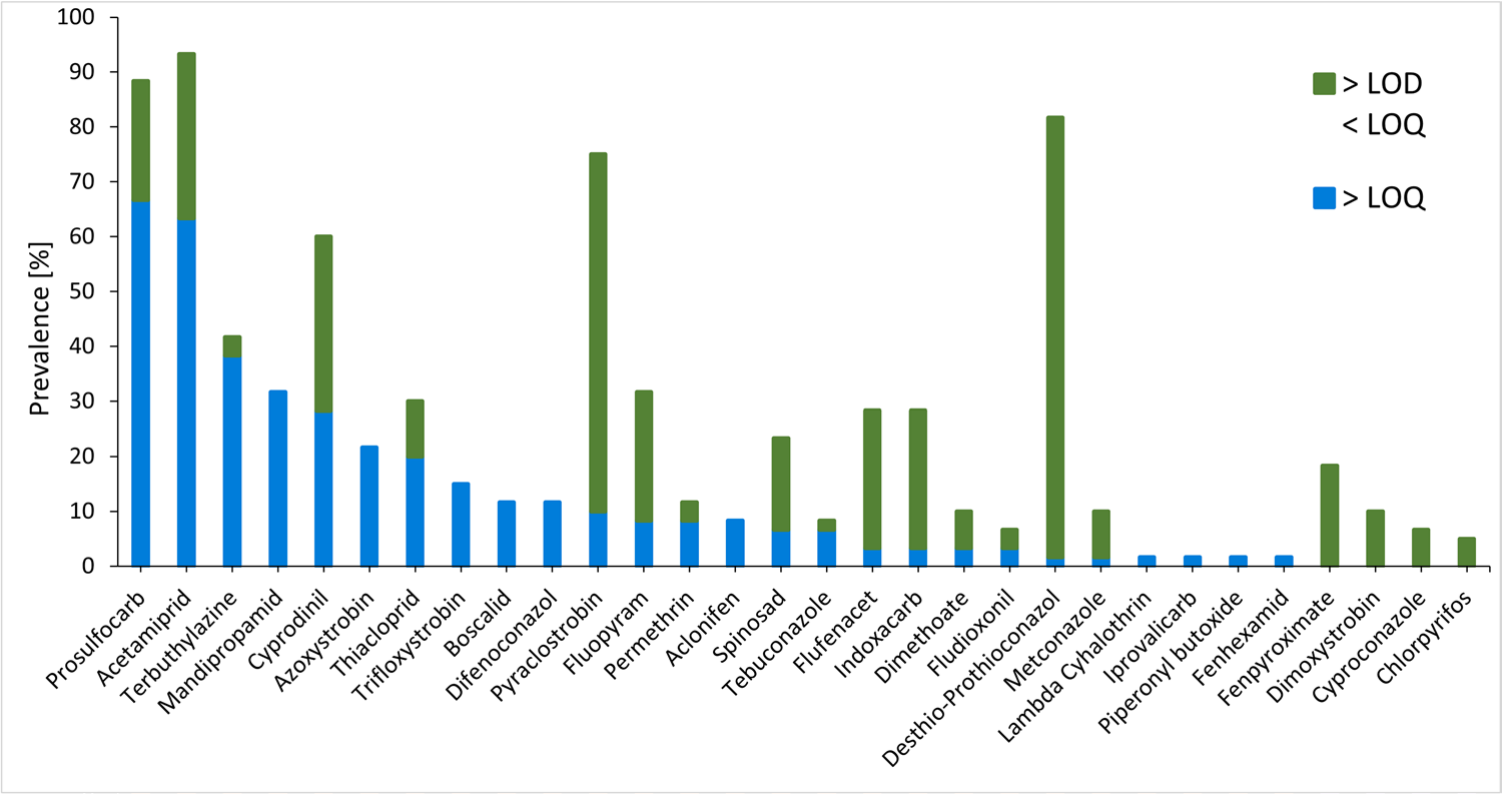
Prevalence of pesticides in bee bread samples. The prevalence of the quantitated pesticides (concentrations > LOQ) is marked in blue, while the prevalence of the pesticides at levels between LOD and LOQ is marked in green. The prevalence (%) is calculated based on a total number of 60 samples

The prevalence of pesticides present in bee bread is shown in Fig. 2. Out of the 51 compounds tested, 30 pesticides were identified (> LOD) at least in one of the samples, while 26 pesticides could be quantitated (> LOQ). The total number of pesticides detected per colony ranged from 11 to 19. Prosulfocarb, acetamiprid, pyraclostrobin, and desthio-prothioconazole were detected in at least 75% of all samples (> LOD). The prevalence of terbuthylazine, mandipropamid, cyprodinil, thiacloprid, and fluopyram was between 30 and 60%, while the prevalence of azoxystrobin, trifloxystrobin, boscalid, difenoconazole, permethrin, spinosad, flufenacet, indoxacarb, dimethoate, metconazole, fenpyroximate, and dimoxystrobin ranged from 10 to 30% (> LOD).

Two neonicotinoid insecticides (acetamiprid and thiacloprid), two herbicides (prosulfocarb and terbuthylazine), and seven fungicides (azoxystrobin, boscalid, cyprodinil, difenoconazole, mandipropamid, pyraclostrobin, and trifloxystrobin) were quantitated at least 10% of the samples (> LOQ) (Fig. 2). Other pesticides (fluopyram, permethrin, aclonifen, spinosad, tebuconazole, flufenacet, indoxacarb, dimethoate, fludioxonil) were quantitated in 3 to 8% of the samples. Yet others (desthio-prothioconazole, metconazole, lambda-cyhalothrin, iprovalicarb, piperonyl butoxide and fenhexamid) were quantitated (> LOQ) in just one of the samples. Additionally, the acaricide fenpyroximate, the insecticide chlorpyrifos, and the fungicides dimoxystrobin and cyproconazole were identified but could not be quantitated.

**Fig. 3:**
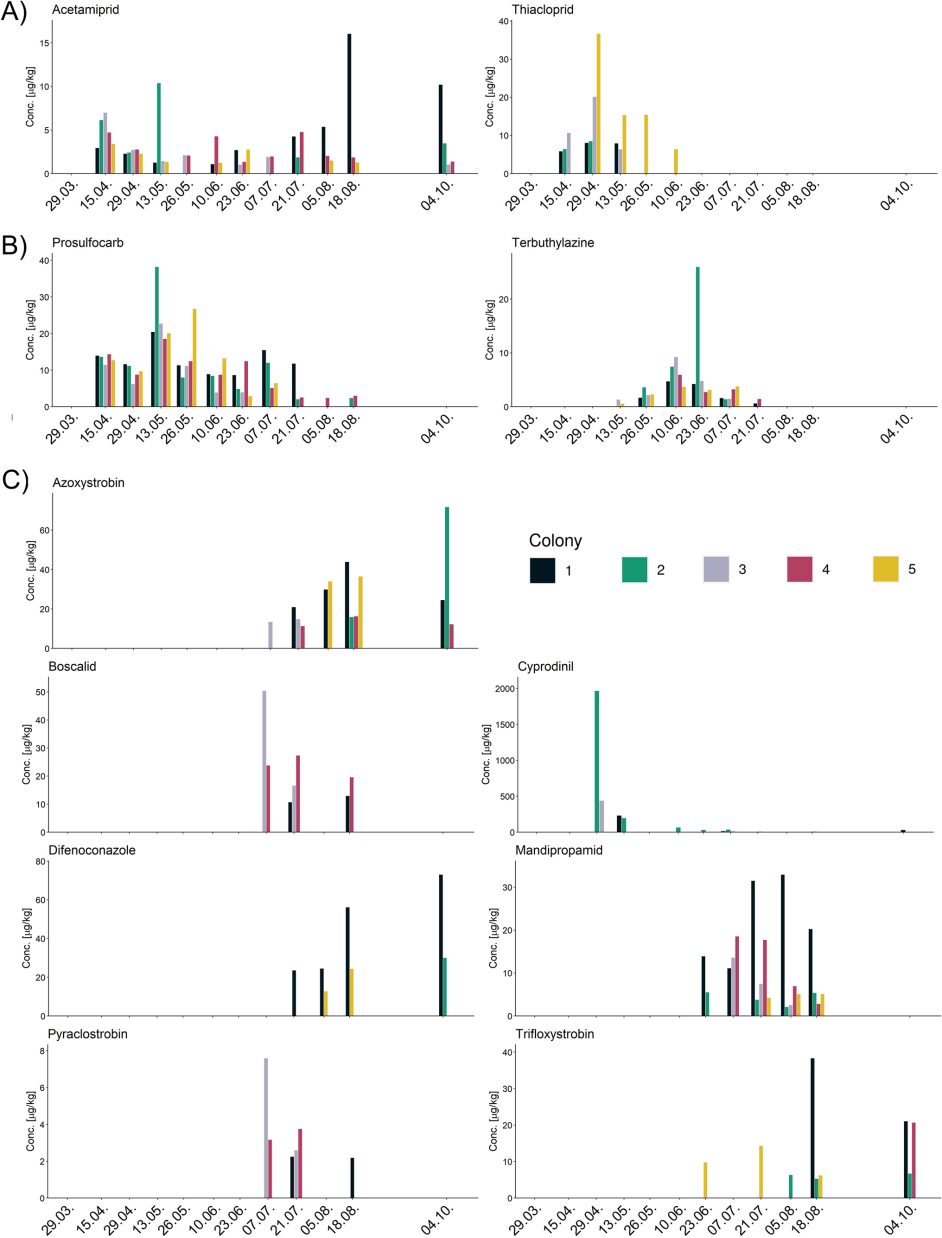
Temporal profiles of the eleven most prevalent pesticides quantifiable in bee bread. **A** insecticides, **B** herbicides, and **C** fungicides. The *x*-axis shows the date in 2022 when a sample was taken

The temporal concentration profiles of the eleven most commonly quantified pesticides are shown in Fig. 3. Depending on the substance, they appeared differently at different points of time. Occurring in April, the earliest quantitated pesticides included the neonicotinoid insecticides acetamiprid and thiacloprid, the herbicide prosulfocarb, and the fungicide cyprodinil. Terbuthylazine was quantitated as early as mid-May 2022. The remaining six pesticides were quantifiable starting from June (mandipropamid, trifloxystrobin) or early July (azoxystrobin, boscalid, difenoconazole, pyraclostrobin) onwards. Five out of the eleven most commonly quantitated pesticides were still present in the last sampling at the beginning of October 2022 (acetamiprid, azoxystrobin, cyprodinil, difenoconazole, and trifloxystrobin).

The occurrence of thiacloprid in bee bread coincided with the blooming of OSR in spring. The appearance of terbuthylazine in bee bread from mid-May to early July occurred before the flowering stage of maize, while azoxystrobin was quantitated during the observed flowering periods of maize and sunflower in late summer (Fig. 3).

**Fig. 4:**
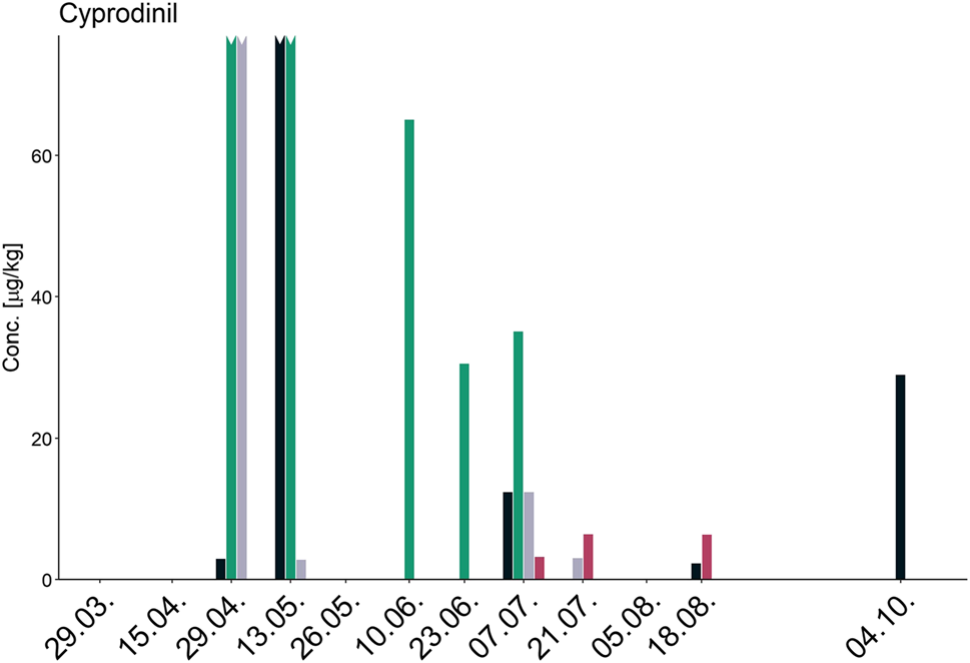
Temporal profile of cyprodinil in bee bread. The *y*-axis was enlarged to display low concentration levels. Four bars (green and grey on 29th April and black and green on 13th May) extend beyond the applied scale as indicated by the jagged top. The colonies are shown in different colours: Black (colony 1), green (colony 2), grey (colony 3), red (colony 4), and yellow (colony 5; not detected). The *x*-axis shows the dates corresponding to when a sample was taken

Even though the colonies were located only a few meters apart at the same apiary, the number of observations of each pesticide varied greatly between colonies (Fig. 3). One insecticide (acetamiprid), two herbicides (prosulfocarb, and terbuthylazine), and two fungicides (azoxystrobin, mandipropamid) were present in all colonies at least once throughout the sampling campaign. The fungicides cyprodinil and trifloxystrobin as well as the insecticide thiacloprid were quantifiable in four colonies, while the fungicides boscalid, difenoconazole, and pyraclostrobin were quantifiable in three out of five colonies (Figs. 3 and 4).

Furthermore, Fig. 3 shows large differences in pesticide levels. The highest residue levels up to a maximum concentration of 1965 μg/kg were measured for cyprodinil in one of the colonies. Azoxystrobin and difenoconazole were quantitated at a maximum of 72 μg/kg and 73 μg/kg, respectively. Lower maximum concentrations ranging from 25 to 50 μg/kg were measured for prosulfocarb, mandipropamid, thiacloprid, trifloxystrobin, terbuthylazine, and boscalid. In contrast, pyraclostrobin and acetamiprid were present at 8 to 16 μg/kg, respectively.

At any given date, the residue levels of pesticides in bee bread were found to differ strongly between the colonies, as the example of cyprodinil on 29th April 2022 shows (Figs. 3 and 4). While residue levels were present in Colony 2 at concentrations near 2000 μg/kg, concentrations in the other colonies’ bee bread were much lower (near 440 μg/kg in Colony 3, near 3 μg/kg in Colony 1), and it was not quantifiable in the remaining two colonies. Similar effects were observed for other pesticides, such as thiacloprid at the same date or terbuthylazine at the end of June 2022. These observations suggest different pollen compositions in bee bread from honey bee colonies located at the same site with the same pollen availability.

## Discussion

A concept was established using honey bees for pesticide monitoring. The study revealed the presence of a large number of pesticides in the bee bread from a Swiss apiary in an agricultural area, suggesting that honey bee colonies are exposed to multiple pesticides. However, little is known about the health effects of such exposure. These circumstances justify using the current concept for monitoring a variety of apiaries to examine regional and transregional differences. In the future, the measured pesticide levels in bee bread as well as seasonal contamination profiles may act as the comparison baseline for future studies.

The presented workflow allowed for the analysis of 51 pesticides, both hydrophilic and lipophilic (log *K*_*ow*_ between −0.1 and 7) with an appropriate method of QuEChERS extraction, UHPLC separation, and subsequent MS/MS quantification. The method was validated with LOQ values ranging from as low as 0.2 up to 10 μg/kg at recoveries ranging from 75 to 125% for 46 out of the 51 pesticides. The seasonal contamination profile of some pesticides correlated with the flowering periods of OSR (thiacloprid) early in the year and of maize (azoxystrobin) or sunflowers (azoxystrobin) in the late-season. This suggests that an adequate sampling should include flowering periods of crops important to honey bees. Furthermore, the variability of pesticide levels in bee bread between the tested colonies suggests that a study needs to include multiple colonies.

The current study was conducted at a single apiary. For an overview of the pesticide exposure of bees in Switzerland, multiple sites should be included in the future. Furthermore, our study gives little information on the crop types responsible for pesticides in bee bread. Ideally, a study would be conducted in a controlled environment where the date of spraying and the crops treated are known. This would allow conclusions to be drawn about the origin of the pesticides in bee bread.

### Sampling time and frequency

Based on the various temporal pesticide profiles in bee bread, it becomes apparent that the time point of sample collection is crucial to the outcome of the study. Some pesticides were quantifiable throughout most of the sampling campaign (e.g., acetamiprid, prosulfocarb). Additional pesticides were quantifiable in early spring (e.g., thiacloprid) coinciding with the flowering period of OSR or early summer (e.g., terbuthylazine) during the seedling stage of maize, while yet others only appear later in the season (e.g., azoxystrobin) coinciding with the flowering periods of maize or sunflower. Thus, if the samples are not taken according to the flowering periods of crops attractive to honey bees, major contamination levels of pesticides in bee bread might be missed. Therefore, to produce contamination profiles across a full crop season, we suggest sampling ideally at least every 2 weeks. If only a smaller number of samples is achievable, we strongly suggest sampling during the flowering periods of major crops attractive to honey bees at the respective study site. However, restricting sampling to full bloom periods of crops attractive to honey bees may underestimate the contamination level, since herbicides or fungicides may be sprayed outside these periods. Additionally, pollen of wild plants might be contaminated by spray drift, thus increasing residual levels of pesticides in bee bread.

### Number of colonies

Since the sampled colonies were located in close proximity, the pollen availability throughout the crop season was the same for all colonies. As described before, pollen composition can vary strongly between colonies at the same study site (Keller et al., 2015; Roncoroni et al., 2021). In the current study, variable pesticide levels might suggest discrepancies in foraging behaviour between colonies of the same apiary. Accordingly, differences in pesticide levels in the bee bread might indicate that forager bees collected dissimilar pollen types with variable contamination levels. For example, thiacloprid was not quantifiable in the bee bread of Colony 4 throughout the whole season, while bee bread from the other colonies showed distinctive contamination levels nearing maximal levels of 40 μg/kg. Consequently, forager bees as well as younger bees of Colony 4 were less exposed to this pesticide compared to the other colonies.

Evidently, our work showed that it is crucial to include a sufficiently large number of colonies per apiary to capture the in-between variabilities, especially for comparison of the contamination levels in transregional studies. We suggest pooling samples from several colonies to decrease the workload and costs, especially if the focus is mainly to determine the prevalence of contaminants in bee bread. However, the pesticide profiles will be smoothed and therefore maximum residue levels might be underestimated.

### Flowering periods of relevant crops and occurrence of pesticides in beebread

The temporal occurrence of thiacloprid in bee bread, a neonicotinoid insecticide formerly designed as spray application in OSR (FSVO 2022), coincided with the observed flowering period of OSR from 5th April to 15th May. Thereby, the first occurrence of thiacloprid in bee bread was detected in mid-April, suggesting an application on OSR. Residues of thiacloprid were still present in the two subsequent samples after the end of the observed flowering period of OSR in the bee bread collected from one of the colonies (Colony 5). Hence, we might have missed some of the prolonged flowering OSR plants or thiacloprid may have remained on the pollen for a few more days. It is reported that the dissipation rate RL_50_ of thiacloprid (i.e., the rate at which it disappears due to processes such as volatilization or hydrolysis) on the plant matrix may be up to 11 days, as found in the pesticide properties database (Lewis & Tzilivakis, 2017; Lewis et al., 2016).

The use of thiacloprid was prohibited by the end of 2021 in the EU and Switzerland as part of phasing out harmful neonicotinoids (Eur-Lex, 2020; Fed-Lex, 2021). The relatively short dissipation rate of 11 days indicates that the residues are unlikely to originate from applications in 2021. Moreover, the first sampling in March did not contain thiacloprid residues, suggesting that the bee bread collected on April 15th consisted of newly collected pollen. Hence, residues from April samples were unlikely from unconsumed bee bread from the previous year. As a consequence, our findings suggest an improper application of thiacloprid in 2022 despite its ban. Subsequent monitoring in the following year would reveal if products containing thiacloprid are still actively applied.

Additionally, for the fungicide azoxystrobin, the findings correspond with the observed flowering period of maize from the beginning of July to August. According to the Swiss Pesticide Database, products containing azoxystrobin are approved for maize cultivations among other crops (FSVO 2022). For maize, azoxystrobin is included in products used as seed dressing with systemic properties (Bartlett et al., 2002). Furthermore, the pesticide is authorized as a spray application for sunflowers, when inflorescence buds between the young leaves are barely visible (stage BBCH 51). Thus, there is no applications of azoxystrobin expected during the blooming cultivation of maize and sunflower due to crop height. We, therefore, hypothesize that during the flowering of maize and sunflower, these pesticides are applied in cultures other than maize or sunflowers and are transported to the flower head of maize or sunflower due to wind drift from neighbouring crops. Additionally, flower heads and pollen of maize might contain azoxystrobin due to systemic transport in the plant (Bartlett et al., 2002).

Terbuthylazine is authorized for use as a herbicide for maize plants with permitted application up to leaf stage BBCH 10 to 16 (FSVO 2022). Terbuthylazine residues were quantifiable in mid-May and June, and thus, the occurrence of the residues correlates with an application during these leaf stages before the flowering of maize. Hence, maize pollen was not yet available. Therefore, we hypothesize that pollen containing terbuthylazine collected by bees during June originates from other cultures or wild plants that were exposed to drift during the spray treatment of the maize seedlings. Previous field studies and models produced by Simon-Delso et al. (2017) have shown that bees are additionally exposed to pesticides applied in crops that are less attractive food sources. They suggest spray drift and/or remobilization of persistent substances during crop rotation.

### Comparison between our, EU, and non-EU studies

Comparison of our current findings from a single apiary with studies performed across Europe and overseas is challenging due to the varying study designs, especially for comparison of the maximal residue levels. Some studies were based on a single sampling time point (Orantes-Bermejo et al., 2015), while others sampled over a longer period but not in detail over a whole crop season (Tong et al., 2018). Additionally, sample numbers, numbers of apiaries, and spatial distribution of the apiaries were very variable. Furthermore, differences in analytical technique may result in different detection and quantification limits, thus affecting the number of pesticides detected.

In Germany, pesticide contamination of bee bread is regularly monitored as part of a national bee monitoring campaign (Deutsches Bienenmonitoring, 2021a, 2021b). In 2020, the study included 128 bee bread samples collected from 118 apiaries during spring (40), summer (83), and fall (5) (Deutsches Bienenmonitoring, 2021b). In 2019, 129 samples of bee bread from 110 apiaries were taken in spring and summer (Deutsches Bienenmonitoring, 2021a). In 2020, 83 pesticides were detected out of 457 analysed pesticides (Deutsches Bienenmonitoring, 2021b). In 2019, the detection rate was 90 out of 455 screened pesticides (Deutsches Bienenmonitoring, 2021a), which included all of the 11 most prevalent pesticides detected in our study. The distribution of the active substance classes was similar to that in our study. The most prevalent were fungicides, especially azoxystrobin and boscalid, followed by herbicides (e.g., prosulfocarb and terbuthylazine) and insecticides, especially thiacloprid. In Germany in 2019, maximum concentrations of the fungicides azoxystrobin, boscalid, prosulfocarb, and trifloxystrobin (max. 482, 339, 106 and 136 μg/kg) as well as the insecticide acetamiprid (max. 51 μg/kg) were found to exceed those of the studied Swiss apiary. On the other hand, the maximum level of the fungicide cyprodinil was 6-fold higher in our study (376 vs. 1965 μg/kg) as compared to Germany in the year 2019 (Deutsches Bienenmonitoring, 2021a). However, the samples of three colonies were pooled, and thus, residue levels of cyprodinil may have been higher in individual samples. Additionally, cyprodinil in bee bread at levels below that in the German study from 2019 have been reported from Italy (8–13 μg/kg) (Porrini et al., 2016) and Poland (< 10 μg/kg) (Kiljanek et al., 2021).

Furthermore, the fungicides azoxystrobin, difenoconazole, pyraclostrobin, and trifloxystrobin have been detected in bee bread from Luxembourg (Beyer et al., 2018), with azoxystrobin, trifloxystrobin, and thiacloprid exceeding maximum concentrations reported in our study. Azoxystrobin (Murcia Morales et al., 2020) and boscalid (Lozano et al., 2019) were reported in Spanish studies at low levels.

The insecticides thiacloprid and acetamiprid have also been found in bee bread samples from France (Daniele et al., 2018) and the Czech Republic (Bokšová et al., 2021) at levels above the maximum concentrations determined in our study. Furthermore, thiacloprid was detected in bee bread from Luxembourg (Beyer et al., 2018).

Overseas, eight of the twelve most prevalent pesticides in our study have been detected in a monitoring campaign performed in the USA by Traynor et al. (2021). They revealed high residue levels of cyprodinil (up to 5800 μg/kg), boscalid (up to 3070 μg/kg), azoxystrobin (up to 1870 μg/kg), and pyraclostrobin (up to 1070 μg/kg), while other pesticides were present at lower concentrations.

### Toxicity to bees

A risk assessment based on the comparison of daily consumption of maximal contaminated bee bread compared to the respective oral acute lethal dose 50 (LD_50_) was performed to obtain an initial assessment of the residue levels in the analysed bee bread. Thereby, a maximum daily pollen uptake of 12 mg per adult nurse bee (Appendix J, Table J1, European Food Safety Authority, 2013) was used. Accordingly, the worst-case scenario of pesticide consumption for an adult nurse bee was determined by multiplying the measured maximum pesticide concentration in bee bread with the maximum daily pollen consumption (Table 5). The respective LD_50_ was divided by the maximal daily uptake per bee to yield the toxicity exposure ratio (TER). TER was calculated for all pesticides, except desthio-prothioconazole (transformation product of prothioconazole) and piperonyl butoxide, for which no oral acute toxicity data was available.

**Table 5:** Risk assessment in relation to the quantitated pesticides in bee bread

Compound	*n*^1^	Max. conc. (μg/kg)	Max. uptake^2^ (μg/bee·day)	LD_50_^3^ (μg/bee)	TER^4^
Prosulfocarb	40	38.2	0.00046	103.4	225,731
Acetamiprid	38	16.0	0.00019	14.53	75,565
Terbuthylazine	23	25.9	0.00031	> 22.6	72,578
Mandipropamid	19	32.9	0.00039	> 200	506,591
Cyprodinil	17	1964.5	0.02357	112.5	4772
Azoxystrobin	13	71.6	0.00086	> 25	29,097
Thiacloprid	12	36.6	0.00044	17.32	39,431
Trifloxystrobin	9	38.3	0.00046	> 200	435,463
Difenoconazol	7	73.0	0.00088	> 177	202,055
Boscalid	7	50.4	0.00060	> 166	274,645
Pyraclostrobin	6	7.6	0.00009	> 110.0	1,208,146
Fluopyram	5	28.0	0.00034	> 102.3	304,464
Permethrin	5	21.0	0.00025	0.13	515
Aclonifen	5	11.4	0.00014	> 107	780,223
Tebuconazole	4	59.7	0.00072	> 83.05	116,003
Spinosad	4	15.2	0.00018	0.057^5^	313
Fludioxonil	2	42.5	0.00051	> 100	196,189
Indoxacarb	2	25.7	0.00031	0.232	752
Flufenacet	2	8.9	0.00011	> 100	941,106
Dimethoate	2	1.2	0.00001	0.1	6872
Lambda Cyhalothrin	1	21.0	0.00025	0.91	3613
Fenhexamid	1	11.9	0.00014	> 102.07	713,374
Desthio-Prothioconazol	1	5.6	0.00007	n.a.^6^	n.a.
Metconazole	1	5.1	0.00006	85	1,398,174
Piperonyl butoxide	1	2.8	0.00003	n.a.^6^	n.a.
Iprovalicarb	1	2.5	0.00003	> 199	6,633,333

^1^Number of positive samples (> LOQ) out of a total of 60 samples

^2^Calculated based on 12 mg of daily pollen consumption by an adult nurse bee and the maximum pesticide residue level quantified

^3^Oral acute LD_50_ values for honey bees (*Apis* spp.) as presented in the Pesticide Properties Database (Lewis et al., 2016)

^4^Toxicity exposure ratio; the ratio of LD_50_ to the maximal uptake of the respective pesticide

^5^As presented in Appendix A of European Food Safety Authority (2018)

^6^No toxicity data available for oral acute LD_50_

The lowest TER was determined for the insecticides spinosad and indoxocarb (TER 313 and 752) as well as for the pyrethroid permethrin (TER 515). While these risk quotients are well above the suggested threshold of 10, sublethal effects may not be disregarded. Recently, exposure to the biopesticide spinosad at environmentally realistic levels has been found to induce alterations in honey bee genes related to energy production (Christen et al., 2019). Similarly, exposure to indoxacarb has been determined to be toxic to honey bees at the recommended application dosage used in the field (Pashte & Patil Shivshankar, 2018).

Regarding the eleven most prevalent pesticides, the determined TER values show that the oral acute LD_50_ values exceed the calculated daily maximum pesticide uptake per bee by an order of magnitude 4 or higher. The summed consumption over the span of 10 days (assuming a nurse bee consuming only maximum contaminated bee bread) only lowers the toxicity exposure by one order of magnitude. Thus, the TER of the isolated concentrations determined in the bee bread is well above the respective oral acute LD_50_, even if such bee bread was consumed over a longer period of time. However, pesticide concentrations may not be evenly distributed between cells. Therefore, individual cells might contain higher pesticide concentrations. Furthermore, little is known about the additive and synergist toxicity of several pesticides, although these effects are suspected to play a greater role in honey bee decline than individual substances (Mullin et al., 2010).

## Conclusion

This study serves as a proof of concept for environmental monitoring. It shows that honey bee colonies in an agricultural environment can be exposed to multiple pesticides. Our work conclusively reveals the advantage of a systematic sampling approach that resulted in a high temporal resolution record from one single apiary across a full crop season. The proposed biweekly sampling and the investigation of individual samples from several colonies has proven necessary to yield meaningful results for the studies site. Based on this systematic workflow, further steps may include sampling at multiple locations within Switzerland over several years, which will enable a comparison between the studied regions and the course of the years. This will allow an overview of pesticide exposure levels of honey bees in Switzerland and can be useful for monitoring the risk reduction measures based on policymaker decisions.

## Data Availability

The data generated during this study is included in this article and is also available from the corresponding author (christina.kast@agroscope.admin.ch). Samples are not available.

## References

[CR1] Bartlett DW, Clough JM, Godwin JR, Hall AA, Hamer M, Parr-Dobrzanski B (2002). The strobilurin fungicides. Pest Manag Sci.

[CR2] Beyer M, Lenouvel A, Guignard C, Eickermann M, Clermont A, Kraus F, Hoffmann L (2018). Pesticide residue profiles in bee bread and pollen samples and the survival of honey bee colonies-a case study from Luxembourg. Environ Sci Pollut Res Int.

[CR3] Deutsches Bienenmonitoring (2021a) Abschlussbericht Zeitraum 2017-2019. Hohenheim University, Germany. Retrieved 18.02.2023 from https://bienenmonitoring.uni-hohenheim.de

[CR4] Deutsches Bienenmonitoring (2021b) Zwischenbericht Zeitraum 2020. Hohenheim University, Germany. Retrieved 18.02.2023 from https://bienenmonitoring.uni-hohenheim.de

[CR5] Bogdanov S (2005). Contaminants of bee products. Apidologie.

[CR6] Bokšová A, Kazda J, Stejskalová M, Šubrt T, Uttl L, Mráz P, Bartoška J (2021). Findings of herbicide and fungicide residues in bee bread. Plant Soil Environ.

[CR7] Carroll MJ, Brown N, Goodall C, Downs AM, Sheenan TH, Anderson KE (2017). Honey bees preferentially consume freshly-stored pollen. PloS One.

[CR8] Christen V, Krebs J, Bunter I, Fent K (2019). Biopesticide spinosad induces transcriptional alterations in genes associated with energy production in honey bees (Apis mellifera) at sublethal concentrations. J Hazard Mater.

[CR9] Daniele G, Giroud B, Jabot C, Vulliet E (2018). Exposure assessment of honey bees through study of hive matrices: analysis of selected pesticide residues in honey bees, beebread, and beeswax from French beehives by LC-MS/MS. Environ Sci Pollut Res Int.

[CR10] Di Pasquale G, Alaux C, Le Conte Y, Odoux JF, Pioz M, Vaissiere BE, Belzunces LP, Decourtye A (2016). Variations in the availability of pollen resources affect honey bee health. PloS One.

[CR11] Environmental Protection Agency (1997). Spinosad.

[CR12] Eur-Lex (2020) Document 32020R0023. Retrieved 18.02.2023 from http://data.europa.eu/eli/reg_impl/2020/23/oj

[CR13] European Food Safety Authority (2013) Guidance on the risk assessment of plant protection products on bees (Apis mellifera, Bombus spp. and solitary bees). EFSA J 11(7). 10.2903/j.efsa.2013.3295 10.2903/j.efsa.2023.7989 PMC10173852 37179655

[CR14] European Food Safety Authority (2018) Peer review of the pesticide risk assessment of the active substance spinosad. EFSA J 16(5). 10.2903/j.efsa.2018.5252 10.2903/j.efsa.2018.5252 PMC7009390 32625896

[CR15] Fed-Lex (2021). Verordnung über das Inverkehrbringen von Pflanzenschutzmitteln.

[CR16] Friedle C, Wallner K, Rosenkranz P, Martens D, Vetter W (2021). Pesticide residues in daily bee pollen samples (April-July) from an intensive agricultural region in Southern Germany. Environ Sci Pollut Res Int.

[CR17] FSVO (2022) Federal Food Safety and Veterinary Office, Switzerland, Pflanzenschutzmittelverzeichnis Retrieved 14.10.2022 from https://www.psm.admin.ch/de/produkte

[CR18] Giroud B, Vauchez A, Vulliet E, Wiest L, Bulete A (2013). Trace level determination of pyrethroid and neonicotinoid insecticides in beebread using acetonitrile-based extraction followed by analysis with ultra-high-performance liquid chromatography-tandem mass spectrometry. J Chromatogr A.

[CR19] Kast C, Kilchenmann V, Charriere JD (2021). Long-term monitoring of lipophilic acaricide residues in commercial Swiss beeswax. Pest Manag Sci.

[CR20] Keller I, Fluri P, Imdorf A (2015). Pollen nutrition and colony development in honey bees: part 1. Bee World.

[CR21] Kiljanek T, Niewiadowska A, Malysiak M, Posyniak A (2021). Miniaturized multiresidue method for determination of 267 pesticides, their metabolites and polychlorinated biphenyls in low mass beebread samples by liquid and gas chromatography coupled with tandem mass spectrometry. Talanta.

[CR22] Lewis K, Tzilivakis J (2017) Development of a data set of pesticide dissipation rates in/on various plant matrices for the Pesticide Properties Database (PPDB). Data 2(3). 10.3390/data2030028

[CR23] Lewis KA, Tzilivakis J, Warner DJ, Green A (2016). An international database for pesticide risk assessments and management. Hum Ecol Risk Assess Int J.

[CR24] Locke B, Forsgren E, de Miranda JR (2014). Increased tolerance and resistance to virus infections: a possible factor in the survival of Varroa destructor-resistant honey bees (Apis mellifera). PloS One.

[CR25] Lozano A, Hernando MD, Ucles S, Hakme E, Fernandez-Alba AR (2019). Identification and measurement of veterinary drug residues in beehive products. Food Chem.

[CR26] Lundin O, Rundlof M, Smith HG, Fries I, Bommarco R (2015). Neonicotinoid insecticides and their impacts on bees: a systematic review of research approaches and identification of knowledge gaps. PloS One.

[CR27] Marti JNG, Kilchenmann V, Kast C (2022). Evaluation of pesticide residues in commercial Swiss beeswax collected in 2019 using ultra-high performance liquid chromatographic analysis. Environ Sci Pollut Res Int.

[CR28] Mullin CA, Frazier M, Frazier JL, Ashcraft S, Simonds R, Vanengelsdorp D, Pettis JS (2010). High levels of miticides and agrochemicals in North American apiaries: implications for honey bee health. PloS One.

[CR29] Murcia Morales M, Gomez Ramos MJ, Parrilla Vazquez P, Diaz Galiano FJ, Garcia Valverde M, Gamiz Lopez V, Manuel Flores J, Fernandez-Alba AR (2020). Distribution of chemical residues in the beehive compartments and their transfer to the honey bee brood. Sci Total Environ.

[CR30] Murcia-Morales M, Heinzen H, Parrilla-Vázquez P, Gómez-Ramos M, Fernández-Alba AR (2022) Presence and distribution of pesticides in apicultural products: a critical appraisal. TrAC Trends Anal Chem 146. 10.1016/j.trac.2021.116506

[CR31] Niell S, Cesio V, Hepperle J, Doerk D, Kirsch L, Kolberg D, Scherbaum E, Anastassiades M, Heinzen H (2014). QuEChERS-based method for the multiresidue analysis of pesticides in beeswax by LC-MS/MS and GCxGC-TOF. J Agric Food Chem.

[CR32] Orantes-Bermejo FJ, Pajuelo AG, Megías MM, Fernández-Píñar CT (2015). Pesticide residues in beeswax and beebread samples collected from honey bee colonies (Apis mellifera L.) in Spain. Possible implications for bee losses. J Apic Res.

[CR33] Pashte V, Patil Shivshankar C (2018). Toxicity and poisoning symptoms of selected insecticides to honey bees (Apis mellifera L.). Arch Biol Sci.

[CR34] Porrini C, Mutinelli F, Bortolotti L, Granato A, Laurenson L, Roberts K, Gallina A, Silvester N, Medrzycki P, Renzi T, Sgolastra F, Lodesani M (2016). The status of honey bee health in Italy: results from the nationwide bee monitoring network. PloS One.

[CR35] PubChem (2022). Dimoxystrobin.

[CR36] PubChem (2022). Fipronil.

[CR37] Requier F, Odoux JF, Tamic T, Moreau N, Henry M, Decourtye A, Bretagnolle V (2015). Honey bee diet in intensive farmland habitats reveals an unexpectedly high flower richness and a major role of weeds. Ecol Appl.

[CR38] Roessink I, van der Steen JJM (2021). Beebread consumption by honey bees is fast: results of a six-week field study. J Apic Res.

[CR39] Roncoroni F, Kilchenmann V, Bieri K, Ritter R, Kast C (2021). Pollensammelverhalten von Völkern am gleichen Standort. Schweizerische Bienen Zeitung.

[CR40] Simon-Delso N, San Martin G, Bruneau E, Delcourt C, Hautier L (2017). The challenges of predicting pesticide exposure of honey bees at landscape level. Sci Rep.

[CR41] Souza Tette PA, Rocha Guidi L, de Abreu Gloria MB, Fernandes C (2016). Pesticides in honey: a review on chromatographic analytical methods. Talanta.

[CR42] Swiss Government (2017) Aktionsplan zur Risikoreduktion und nachhaltigen Anwendung von Pflanzenschutzmitteln. Retrieved 29.11.2022 from https://www.blw.admin.ch/blw/de/home/nachhaltige-produktion/pflanzenschutz/aktionsplan.html

[CR43] Swiss Government (2021) Bundesgesetz über die Verminderung der Risiken durch den Einsatz von Pestiziden. Retrieved 29.11.2022 from https://www.bk.admin.ch/ch/d/pore/rf/cr/2021/20210841.html

[CR44] Tong Z, Duan J, Wu Y, Liu Q, He Q, Shi Y, Yu L, Cao H (2018). A survey of multiple pesticide residues in pollen and beebread collected in China. Sci Total Environ.

[CR45] Traynor KS, Tosi S, Rennich K, Steinhauer N, Forsgren E, Rose R, Kunkel G, Madella S, Lopez D, Eversole H, Fahey R, Pettis J, Evans JD, Dennis, v. (2021). Pesticides in honey bee colonies: establishing a baseline for real world exposure over seven years in the USA. Environ Pollut.

[CR46] van der Steen JJM (2016) The colony of the honey bee (Apis Mellifera L) as a bio-sampler for pllutants and plant pathogens. Wageningen University. 10.18174/375348

[CR47] Visscher PK, Seeley TD (1982) Foraging strategy of honey bee colonies in a temperate deciduous forest. Ecology 63(6). 10.2307/1940121

[CR48] Wilmart O, Legreve A, Scippo ML, Reybroeck W, Urbain B, de Graaf DC, Spanoghe P, Delahaut P, Saegerman C (2021). Honey bee exposure scenarios to selected residues through contaminated beeswax. Sci Total Environ.

